# Cognitive Impairment and Depressive Symptoms in a Patient With Obstructive Sleep Apnea: Full Recovery After CPAP Treatment

**DOI:** 10.7759/cureus.12152

**Published:** 2020-12-18

**Authors:** Margarida V Araújo, Andreia Norton

**Affiliations:** 1 Psychiatry, Hospital Magalhães Lemos, Oporto, PRT

**Keywords:** depressive symptoms, cognitive impairment, obstructive sleep apnea syndrome, continuous positive airway pressure (cpap)

## Abstract

Obstructive sleep apnea syndrome (OSA) is associated with neuropsychiatric symptoms, including cognitive impairment and depression. It is important to be aware of this association since these comorbid symptoms may be misdiagnosed as a primary psychiatric condition.

We report a case of a 60-year-old man with depressive symptoms and cognitive impairment, with important deficits in memory and great functional impairment. There was no response to many antidepressant trials and, later, he underwent polysomnography and was diagnosed with severe OSA. The patient started treatment with continuous positive airway pressure (CPAP) and showed progressive improvement in depressive and cognitive symptoms. During one year of follow-up, there was no recurrence of psychiatric symptoms and the patient was able to stop antidepressants and to recover his functionality.

This case highlights the importance of searching for OSA signals when assessing patients with depressive and cognitive symptoms, since they may improve with OSA adequate treatment.

## Introduction

Obstructive sleep apnea syndrome (OSA) is a chronic condition characterized by upper airway obstruction during sleep that leads to sleep fragmentation and intermittent hypoxemia [1].

Being one of the most prevalent chronic respiratory diseases, it affects 5-15% of the general population and its prevalence increases with age up to 65 years, at least [2]. Many patients with OSA remain undiagnosed and untreated, in part due to a lack of awareness of the disorder and the presence of non-specific symptoms, such as fatigue or sleepiness, that may be attributed to other causes [3].

Besides the well-known cardiovascular and metabolic disorders, OSA has also been associated with cognitive impairment, depression, anxiety, and other neuropsychiatric symptoms [4]. It is of crucial importance to be aware of this association since these comorbid symptoms may be misdiagnosed as a primary psychiatric condition and require adequate treatment, as we report in this case.

## Case presentation

A 60-year-old man, who completed 11th grade, was referred to our Psychiatry outpatient department for depressive symptoms, with prominent apathy, lack of initiation, psychomotor retardation, daytime sleepiness, and cognitive impairment. These symptoms were present, continually, at least, for five years and were associated with great functional impairment. The patient stayed at home, in bed, the majority of the day and had to be helped in many daily activities such as preparing meals, handling the accounts, and shopping. He was helped by his sister and brother-in-law. During our consultations, he didn’t report any problems related to sleep besides daytime sleepiness, and we did not have any collateral information about sleep events during the night.

The patient had a medical history of arterial hypertension, diabetes mellitus, obesity, with a body mass index of 36.5, and interstitial lung disease. He had no previous history of psychiatric disorders, including other depressive or manic episodes.

During our medical follow-up, which had a duration of four years, the patient has undergone extensive examination. He was evaluated by Neurologists and acute neurological disorder was excluded. Brain MRI showed signs of ischemic leukoencephalopathy, probably related to arterial hypertension. Electroencephalogram was normal and cerebrospinal fluid (CSF) test results didn’t reveal any abnormality. Blood tests including full blood count, renal, liver and thyroid functions, electrolytes, vitamin B12, and folate were within the normal range. HIV and syphilis tests were negative. Formal cognitive assessment revealed impairment of attention, memory, and processing speed, which was interpreted in the context of depression. In Addenbrooke’s Cognitive Examination-Revised, he scored 81 out of 100. After this investigation, it was assumed a depressive episode and the patient was treated with different antidepressants (sertraline 100mg, duloxetine 60mg, bupropion 150mg, fluoxetine 40mg) for a substantial time with poor response. Based on the lack of response to antidepressants, the medical history of obesity, and the existence of symptoms such as daytime sleepiness and cognitive impairment, an OSA diagnosis was then hypothesized. The patient underwent polysomnography and was referred to a specific consultation with a Pulmonologist. He was diagnosed with severe OSA, with an apnea-hypopnea index of 52, and started treatment with continuous positive airway pressure (CPAP). After he started the CPAP, he showed progressive improvement in depressive and cognitive symptoms. During one year of follow-up after initiating CPAP, there was no recurrence of psychiatric symptoms and the patient was active, with no psychomotor retardation and was able to stop antidepressants and recover his functionality.

## Discussion

We report a challenging case, as the patient presented to our service with depressive symptoms and cognitive deficits for many years, with functional impairment, and had no response to many antidepressant trials during follow-up. The lack of collateral information added difficulties in exploring the existence of nocturnal events, such as snoring or observed episodes of stopped breathing, which would be useful to inform about the possibility of a sleep disorder sooner. After many consultations, a diagnosis of OSA was hypothesized and the patient showed great recovery in neuropsychiatric symptoms after initiating CPAP treatment.

Literature shows that OSA is associated with cognitive dysfunction and studies have identified attention, episodic memory, working memory and executive functions as the most affected cognitive domains. It appears that the associated sleep fragmentation and nocturnal hypoxemia, and not the recurrence of apnea per se, are important mediators of cognitive impairment in patients with OSA [5-7]. The severity of sleep fragmentation appears to be linked to deficits in attention and hypoxemia appears to be associated with impairment in global cognitive function [6]. Passivity, loss of facial expression, and psychomotor retardation may be noticed both in OSA and depression, which makes it difficult to distinguish between these two conditions. Nevertheless, several studies demonstrated an increased risk of developing depression in OSA patients and depression may be related to architectural changes in sleep [8,9].

In relation to treatment, effective CPAP treatment appears to improve cognitive function and adherence to treatment is crucial for this improvement [10,11]. However, even with effective CPAP treatment, in severe cases, patients may not experience a complete reversal of some cognitive domains, which did not happen in our case [6]. A systematic review and meta-analysis found that CPAP treatment in OSA patients improves depressive symptoms, with the greatest benefit occurring in patients with more severe depressive symptoms [12].

In our case, the patient showed great improvement in cognitive and depressive symptoms after initiating CPAP, which led us to believe that those symptoms were secondary to OSA. Another hypothesis equated for this recovery would be the natural history of a depressive episode, with spontaneous recovery after some months, even without treatment. However, this patient was symptomatic for many years and had no response to many antidepressants. Besides, the great recovery presented after he initiated the CPAP treatment makes this hypothesis less probable. Another important question relates to cognitive impairment and signs of ischemic leukoencephalopathy in Brain MRI. Could these cognitive symptoms be associated with vascular brain disorder? We cannot assure that some of the symptoms presented in this case were not related to that. Nevertheless, the improvement after CPAP leads us to believe that, at least in part, the symptoms presented were related to OSA pathophysiology.

## Conclusions

This case report highlights the importance of considering other diagnoses when accessing patients with neuropsychiatric symptoms. In particular, as patients with OSA may present with depressive and cognitive symptoms, which may mimic a primary psychiatric condition, it is of crucial importance to search for OSA signals when accessing those patients. Additionally, it is important to keep in mind that adequate OSA treatment may improve psychiatric symptoms in some patients and limit the exposure to other medications that have no major impact on the patient's psychiatric status. 
